# Classification of aggressive and classic mantle cell lymphomas using synchrotron Fourier Transform Infrared microspectroscopy

**DOI:** 10.1038/s41598-019-49326-3

**Published:** 2019-09-06

**Authors:** Magdalena Kolodziej, Dorota Jesionek-Kupnicka, Marcin Braun, Vitaliy Atamanyuk, Sylwia Sloniec, Jozef Cebulski, Marian Cholewa, Janusz Kopczynski, Philip Heraud, Mark J. Tobin, Jitraporn Vongsvivut, Izabela Zawlik

**Affiliations:** 1 Faculty of Medicine, University of Rzeszow, Rzeszow, Poland; 2 Department of Pathology, Chair of Oncology, Medical University of Lodz, Lodz, Poland; 3 Postgraduate School of Molecular Medicine, Medical University of Warsaw, Warsaw, Poland; 4 Faculty of Mathematics and Natural Sciences, University of Rzeszow, Rzeszow, Poland; 5 Department of Pathology, Holy Cross Cancer Center, Kielce, Poland; 6 Department of Microbiology & Monash Biomedical Discovery Institute, Faculty of Medicine, Nursing and Health Sciences, Monash University, Victoria, Australia; 7 Centre for Biospectroscopy, School of Chemistry, Monash University, Clayton, Australia; 8 Australian Synchrotron, ANSTO, Clayton, Victoria Australia; 9 Centre for Innovative Research in Medical and Natural Sciences, Faculty of Medicine, University of Rzeszow, Rzeszow, Poland; 10 Department of Genetics, Institution of Experimental and Clinical Medicine, University of Rzeszow, Rzeszow, Poland

**Keywords:** B-cell lymphoma, Infrared spectroscopy

## Abstract

Mantle cell lymphoma (MCL) is regarded as an incurable neoplasm, even to the novel drug strategies. It is known MCL has two morphological variants- classic and aggressive. Aggressive MCL is characterized by a higher mitotic index and proliferation rate, and poor overall survival in comparison to classic subtype. The insight into the detailed biochemical composition of MCL is crucial in the further development of diagnostic and treatment guidelines for MCL patients; therefore Synchrotron radiation Fourier Transform Infrared (S-FTIR) microspectroscopy combined with Principal Component Analysis (PCA) was used. The major spectral differences were observed in proteins and nucleic acids content, revealing a classification scheme of classic and aggressive MCLs. The results obtained suggest that FTIR microspectroscopy has reflected the histopathological discrimination of both MCL subtypes.

## Introduction

Mantle cell lymphoma (MCL) is a mature B-cell lymphoma, which originates from the inner mantle zone and is characterised by elevated expression of cyclin D1 as a result of the 11q13 translocation^1^. MCL diagnosis is based on standard histopathological examination complemented essentially by immunohistochemical staining for CD20, CD5, cyclin D1 and Ki67 and frequently supplemented by broad immunohistochemical panels including SOX11, TP53, p16, C-MYC, molecular analysis of 11q13 translocation and inclusion of clinical parameters, such as lactate dehydrogenase levels and white blood cell counts^2,3^.

MCL is regarded as an incurable neoplasm, and is resistant to novel drug strategies^2,4^; therefore, there is an emerging need for unravelling the biology of this malignancy. It is already known that MCL is a heterogeneous disease and distinct morphological variants have been described. These encompass classic and aggressive (blastoid and pleomorphic) mantle cell lymphomas^2–4^. The classic MCL is composed mainly of small-to-medium sized lymphocytic cells with moderate features of malignancy and displays mantle zone, nodular, or diffuse architecture. The aggressive MCLs, on the other hand, show high malignant features (resembling lymphoblasts in blastoid variant and resembling large, heterogeneous, anaplastic cells in pleomorphic variants). The classic MCL is characterised by lower mitotic index and lower proliferation rate (Ki67/MiB-1 index) in comparison to both aggressive subtypes^5^. The aggressive MCLs have more frequently poor prognostic mutations in TP53 and CDKN2A/B genes in comparison to the classic MCLs^6,7^. Finally, the aggressive MCLs are characterised by a poorer progression-free and overall survival, when compared to classic variants and according to current guidelines aggressive MCL patients are allocated into high-risk groups^2,5^.

Although the MCLs classification is well-established with histopathological assessment, the insight into the molecular/biochemical information would be invaluable for a better description of both entities and would be a fundamental proof for the validity of their classification.

Fourier Transform Infrared (FTIR) microspectroscopy offers a novel approach for the assessment of biochemical changes such as healthy and cancerous tissue differentiation and the determination of cancer subtypes without the use of any additional reagents^8,9^. Synchrotron radiation (SR) sources provide bright, broadband infrared light, enabling the analysis of micron-sized samples with higher than the conventional signal to noise than is possible with conventional IR sources^10^. The purpose of the current study was to determine differences between classic and aggressive mantle cell lymphomas using S-FTIR microspectroscopy combined with PCA analysis of the acquired spectroscopic data.

## Results

**Figure 1:**
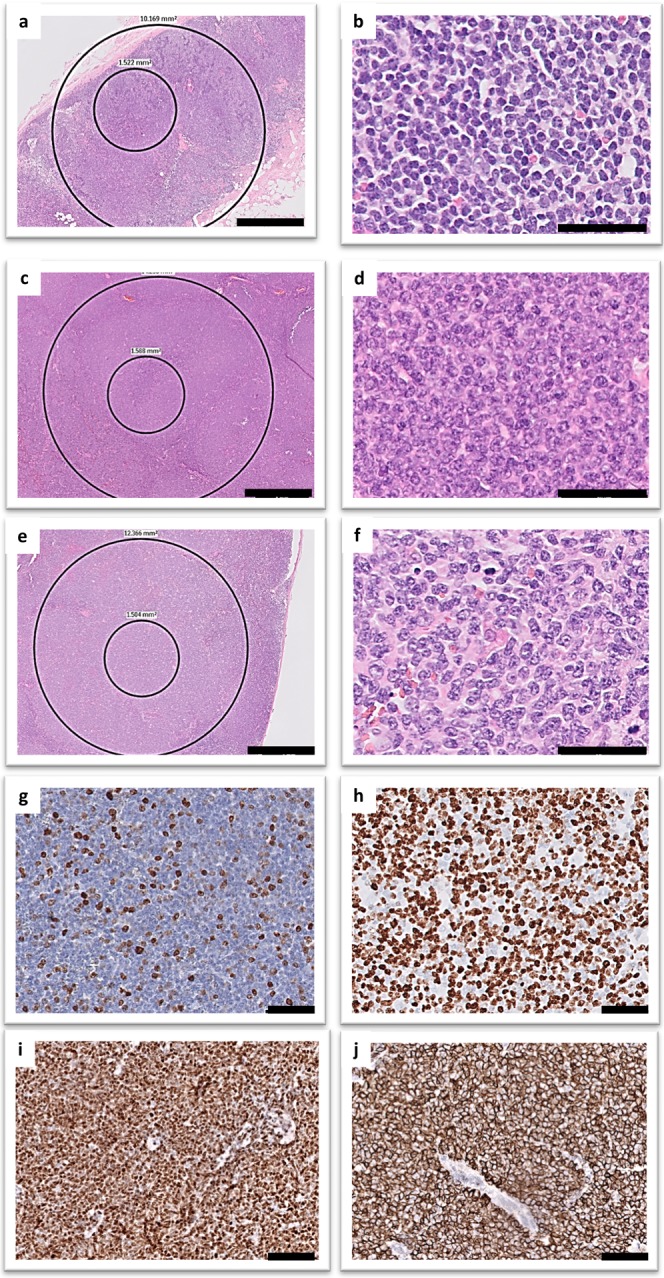
Haematoxylin/eosin histopathological images of representative control and lymphoma tissues. (**a,b**) non-neoplastic control lymph node, (**c,d**) classic mantle cell lymphoma, (**e,f**) and aggressive mantle cell lymphoma. Ki67 proliferation index in (**g**) classic and (**h**) aggressive MCL. All MCL cases were positive for cyclin D1 (**i**) and CD5 (**j**). Scale bars: 1 mm in (**a,c,e**) and 50 um in (**b,d,f,g–j**).

Histopathological micrographs of control and malignant lymph node tissues, classic and aggressive MCL, are shown in Fig. 1.

### FTIR spectral description

**Figure 2:**
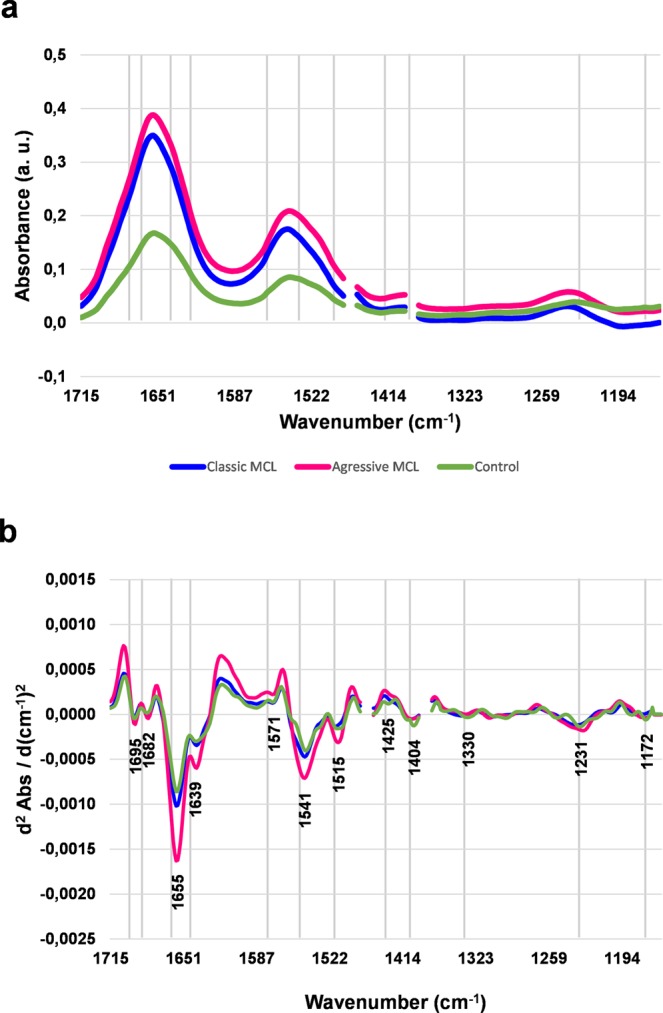
Comparisons of the average absorbance (**a**) and EMSC-corrected second derivative (**b**) spectra of the healthy control, classcic MCL and aggressive MCL with assigned minima.

Representative absorbance (A) and Extended Multiplicative Scattering Corrected second derivative (B) average spectra of the healthy control and two MCL subtypes are presented in Fig. 2. Since the visual inspection of peak positions on raw spectra was difficult, spectra were transformed into the second derivative to enhance the features of overlapping bands (Fig. 2b). The second derivative spectrum gives a negative value for every band located in the absorbance spectrum and allows for more accurate identification of individual peaks in complex spectra. The averaged spectra of every patient included in the further analysis can be found in Supplementary Figs S1–S4.

The absorbance minima determined for all tissues in the protein region are vibrations of amide I (1700–1630 cm^−1^) and amide II (1580–1500 cm^−1^) functional groups^10–12^. The peaks localised at 1695 cm^−1^, 1682 cm^−1^ and 1639 cm^−1^ are characteristic for aggregated β-sheet, β-turn, and β-sheet structures, respectively^11,13^. Typically, the minimum found at 1655 cm^−1^ is attributed to α-helix structures of amide I^13^. The (N–H) bending coupled to (C–N) symmetric stretching vibrations assigned to amide II are localised at 1571 cm^−1^ and 1541 cm^−1^ ^12,14^. Of note is a peak found at 1515 cm^−1^, typically attributed to (C–H) bending vibrations of tyrosine^15^. Of interest are absorbance intensity changes observed for malignant tissues. Both classic and aggressive MCL represents an increase in absorbance intensity noted in peak attributed to α-helix (1655 cm^−1^) structure of amide I, more pronounced in aggressive MCL (Fig. 2b). Absorbance intensity increase noticed in aggressive subtype has also been observed for minima attributed to amide I β-sheet (1639 cm^−1^), amide II (1541 cm^−1^) and tyrosine (1515 cm^−1^) (Fig. 2b). It was already reported that features associated with an aggressive clinical course of MCL included overexpression of the p53 protein^6^. This protein plays an important role in the regulatory control of the cell cycle and its mutations have been associated with the progression to more aggressive forms of the disease^6,16^.

The absorbance minima found at 1425 cm^−1^ and 1330 cm^−1^ are typically responsible for asymmetric and symmetric CH_3_ and CH_2_ bending vibrations of lipids and proteins^8,10^. The peak localised at 1404 cm^−1^ is responsible for (CH_3_) bending vibrations of proteins^13^.

The other prominent peaks occur in the lower wavenumber region. The peak found in control tissue at 1231 cm^−1^, assignable to asymmetric stretching vibrations of ${{\rm{PO}}}_{2}^{-}$ in DNA^17^ is shifted towards higher wavenumber by 3 cm^−1^ and 7 cm^−1^ in classic and aggressive MCL respectively. Moreover, the absorbance intensity of this peak is increased in aggressive MCL, which coincide with available knowledge about cyclin D1 overexpression^18^. The absorbance intensity of minimum localised at 1172 cm^−1^, attributed to symmetric stretching vibrations of ${{\rm{PO}}}_{2}^{-}$ in DNA^17^ is slightly decreased in both MCL subtypes, indicating DNA fragmentation associated with apoptotic cell death^19^. The detailed assignments of the minima found in 2^nd^ derivative spectra are shown in Table 1.

**Table 1:** Summary of mean values of wavenumbers (cm^−1^) seen in FTIR spectra of classic and aggressive MCL and control tissue.

Band position (cm^−1^)	Vibrational mode	References
1695	Amide I: aggregated β-sheet	^13^
1682	Amide I: β-turn structure	^11^
1655	Amide I: α-helix	^13^
1639	Amide I: β-sheet structure	^11^
1571	δ_as_(N–H) and *ν*_s_(C–N) stretch in amide II	^12^
1541	Amide II: δ(N–H) coupled to *ν*(C–N) vibrational modet AAmide II: perpendicular modes of α-helix and parallel-chain *β*-sheet	^10, 14^
1515	δ(C–H) from tyrosine	^15^
1425	δ_as_(CH_3_) and δ_as_(CH_2_) of lipids and proteins	^8^
1404	δ(CH_3_) of proteins	^13^
1330	δ_s_(CH_3_) and δ_s_(CH_2_) of lipids and proteins	^10^
1231	*ν*_as_ ${(\text{PO}}_{2}^{-})$ of DNA	^17^
1172	*ν*_s_ ${(\text{PO}}_{2}^{-})$ of DNA	^17^

Abbreviations: *ν*_s_ – symmetric stretch; *ν*_as_ – asymmetric stretch; δ_s_ – symmetric in-plane deformation (bend); δ_as_ – asymmetric in-plane deformation (bend).

### Principal component analysis

**Figure 3:**
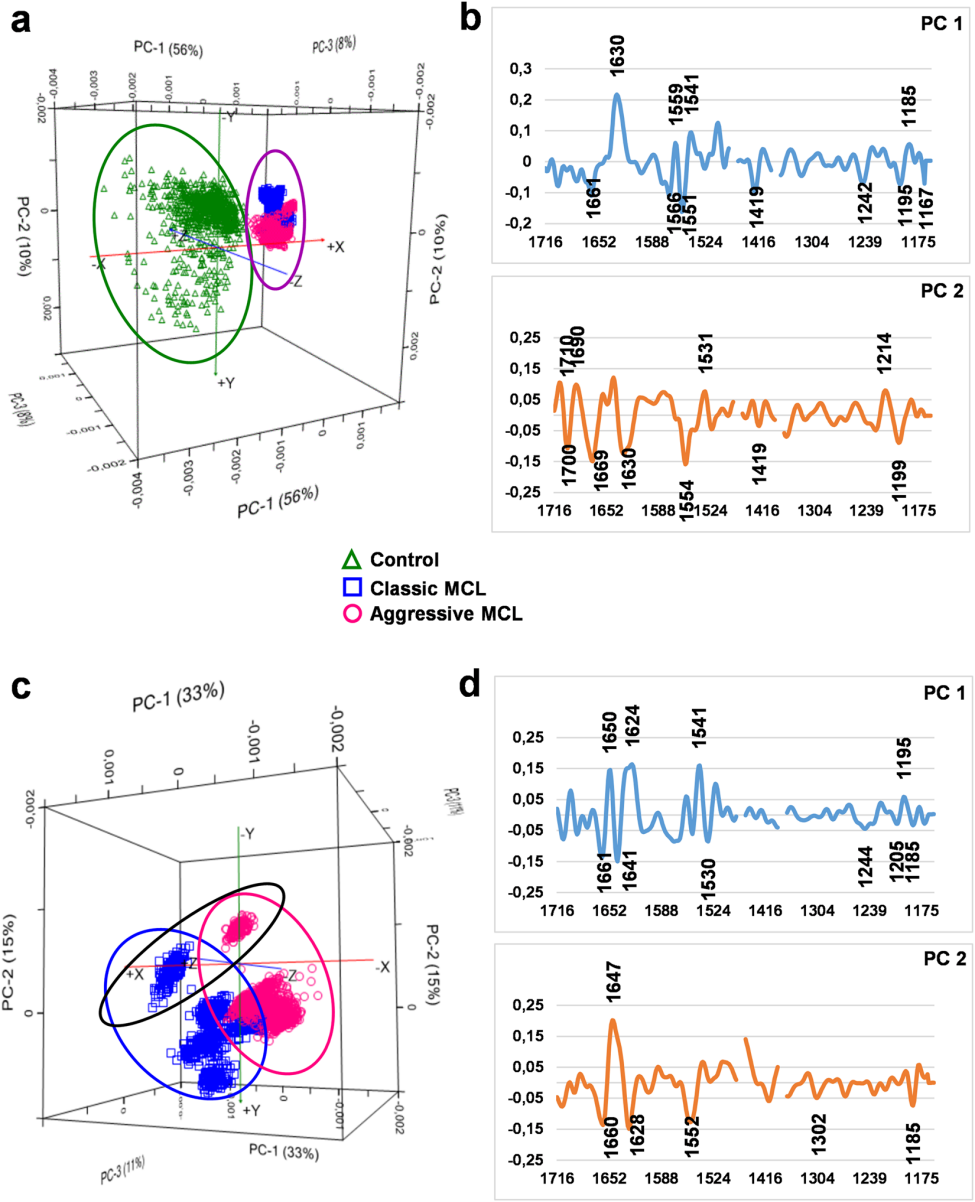
PCA scores (left) and loadings (right) plots showing projections against the first 3 PCs that explain the majority of the spectral variation. (**a,b**) Control (green ellipse) and both malignant tissues (purple ellipse) spectral datasets, and (**c,d**) classcic (blue ellipse) and aggressive (pink ellipse) MCL tissues, alone. Black ellipse indicates dead patients.

The PCA results were obtained with three spectral ranges 1720–1495 cm^−1^, 1440–1400 cm^−1^ and 1360–1160 cm^−1^ covering spectral features characteristic for proteins, lipids, carbohydrates and nucleic acids functional groups. Initially PCA was performed to differentiate MCL tissues from healthy control and results are presented on Fig. 3a,b. The PC loading plots show the amide band region attributable to proteins (1700–1500 cm^−1^) was heavily loaded for PC1 revealing separation of healthy control from both malignant tissues with 56% explained variance (green ellipse, Fig. 3a). Spectra of control can be distinguished from MCL tissues by having negative PC1 scores (Fig. 3a), which can be explained by strong positive loading observed at 1630 cm^−1^ attributed to β-sheet structures of amide I functional group (Fig. 3b). Positive loadings at 1559 cm^−1^ and 1541 cm^−1^, attributable to amide II protein conformers, also separated healthy control cluster from malignant tissues. Moreover, both cancer tissues spectra are separated by positive PC1 scores (purple ellipse, Fig. 3a), explained by strong negative loadings indicated at 1661 cm^−1^ (amide I)^14^, 1566 cm^−1^ ((COO–) asymmetric stretching vibrations of amide II)^20^ and 1551 cm^−1^ (amide II α-helix structures)^20^ (Fig. 3b). These outcomes suggest that the amide I and II structures are most responsible for discrimination of healthy control from lymphoma tissues, which confirms the conclusions drawn from the examination of the average spectra (Fig. 2). Other differences with an impact on classification involve the negative loadings from rocking vibrations of CH_2_ of distributed cis-olefins (1419 cm^−1^) and C=O stretching from polysaccharides (1195 cm^−1^, 1167 cm^−1^) as well as positively loaded peak responsible for symmetric stretching vibrations of ${{\rm{PO}}}_{2}^{-}$ (1185 cm^−1^) (Fig. 3b). The PC2 loading plot reveals components mainly responsible for healthy control spectral data dispersion.

Subsequently, PCA was performed including only the lymphoma tissue spectral datasets. Results presented in Fig. 3c reveals separation of spectral clusters of classic (blue ellipse) and aggressive (pink ellipse) MCL. Classic MCL distinction is explained by negative loadings at 1661 cm^−1^, 1641 cm^−1^ and 1530 cm^−1^ assignable to amide I and II protein conformers (Fig. 3d). Aggressive MCL cluster can be distinguished by positive loadings observed at 1650 cm^−1^, 1624 cm^−1^ and 1541 cm^−1^. This outcomes clearly corresponds to changes in absorbance intensity of protein moiety of described average spectra: in the aggressive subtype protein level is higher than in classic MCL (Fig. 2). Of interest is a PC2 positive loading observed at 1647 cm^−1^ and assigned to α-helix amide I structures. This loading seems to be responsible for distinction of spectra obtained from two patients, for whom the treatment was not successful (black ellipse, Fig. 3c).

## Discussion

This research has demonstrated that the histopathological subtyping of MCL into classic and aggressive forms has its strong background in the biochemical landscape of both subtypes. It should be emphasised that this is the first study which has reported the combination of S-FTIR and PCA analysis for the assessment of MCL subtypes. We previously reported the usefulness of the presented approach in distinction of lung cancer subtypes and estimation of chemotherapy efficacy in breast cancer^8,9^.

Our present results showed an absorbance increase in peaks attributed to amide I, amide II and nucleic acids noticed in both malignant tissues, much more pronounced in aggressive MCL. The shift of wavenumber was observed for PO_2_ asymmetric stretching vibrations corresponding to DNA. PCA loadings revealed amide I rich in β-sheet structures and amide II bands mainly differentiate two MCLs. These results correlate with available knowledge about proteins overexpression in the aggressive MCL subtypes.

In conclusion, classic and aggressive MCL are distinct biological and biochemical entities. FTIR spectroscopy was a sensitive tool for the distinction of the biochemical composition of both subtypes and this knowledge may be beneficial for understanding the biology of MCL.

## Methods

### Material

The study was conducted under the Institutional Review Board (Protocol No. KBET/6/06/2014) from June 2014 at the University of Rzeszow. The experimental protocols used in this study were approved by the institutional ethics committees (IECs) of the University of Rzeszow and were carried out in accordance with the approved guidelines. Informed consent was obtained from all subjects.

The research was conducted on formalin-fixed paraffin embedded (FFPE) tissue samples that were prepared according to standard protocols. The material was obtained from 18 patients with mantle cell lymphoma. Patients were hospitalised in the Holy Cross Cancer Center in Kielce and examined in the Department of Hematology of Medical University in Lodz between 2009–2018, and treated for MCL with customised chemotherapy. All patients were of white race, eight males, nine females and one unknown. Nine patients were diagnosed with classic MCL and nine patients with aggressive MCL. Clinicopathological characteristics of all MCL patients are presented in Table 2.

**Table 2:** Cliniopathological characteristics of patients with mantle cell lymphoma.

Case No.	Age	Sex	Histological type	Cyclin D1	Ki 67 index	First-line treatment	Relapse	Next-line treatment	Long term follow-up
50	ND	F	blastoid/pleomorphic	positive	90	RCHOP	ND	ND	ND
6	82	M	blastoid/pleomorphic	positive	90	RCHOP	yes	R-Benda	Death
7	59	F	blastoid/pleomorphic	negative	100	RCHOP	yes	R-Benda	Death
22	ND	ND	blastoid/pleomorphic	positive	80	ND	ND	ND	ND
27	70	M	blastoid/pleomorphic	positive	70–80	RCHOP	yes	R-Benda	Alive
4	76	F	classic	positive	40	RCVP	yes	R-Benda	Alive
8	70	F	classic	positive	20	RCHOP	no	no	Alive
18	67	M	classic	positive	30	ND	ND	ND	ND
21	70	F	classic	positive	20–40	RCP	yes	R-Benda	Alive
23	86	M	classic	positive	20	ND	ND	ND	ND
25	75	F	classic	positive	40–50	RCHOP	yes	Benda	Alive
28	68	M	classic	positive	50	RCHOP	no	no	Alive
30	86	M	classic	positive	30–50	R-Benda	no	no	Death
32	75	F	classic	positive	40–50	RCHOP	no	no	Death
36	88	F	blastoid/pleomorphic	positive	40	COP	ND	ND	Death
37	83	M	blastoid/pleomorphic	positive	80	RCOP/RCP	ND	ND	Alive
39	70	F	blastoid/pleomorphic	positive	50	RCHOP	ND	ND	Death
40	76	M	blastoid/pleomorphic	positive	90	RCHOP	ND	ND	Death

(Abbreviations: RCHOP = Rituximab, Cyclophosphamide, Doxorubicin (Hydroxydaunomycin), Vincristine (Oncovin), Prednisolone; RCVP = Rituximab, Cyclophosphamide, Vincristine, Prednisolone; RCP = Rituximab, Cyclophosphamide, Prednisolone; COP = Cyclophosphamide, Vincristine (Oncovin), Prednisolone; R-Benda = Rituximab, Bendamustine; ND = no data).

### Pathological diagnostic approach

The standard panel of antibodies examined in patients with MCL covered CD20, CD3, BCL2, BCL6, MYC, CD10, MUM1, Ki67, cyclin D1, SOX11, TdT, CD5, CD38 and PAX5 (BSAP). The antibodies clones along with the manufacturer are named in Table 3.

**Table 3:** The list of antibodies used in the immunohistochemical analysis.

Antibody	Clone	Company
BCL2	124	DAKO
BCL6	PG-B6p	DAKO
BSAP (PAX-5)	DAK-Pax5	DAKO
CD3	Polyclonal rabbit	DAKO
CD5	4C7	DAKO
CD10	56C6	DAKO
CD20	L26	DAKO
CD38	SP149	Cell Marque
Cyclin D1	EP12	DAKO
MYC	Y69	VENTANA
Ki-67	MIB-1	DAKO
MUM-1	MUM1p	DAKO
SOX-11	MRQ-58	Cell Marque
TdT	EP266	DAKO

Immunohistochemical analysis used monoclonal antibodies [FLEX Monoclonal Mouse Anti-Human, Ready-to-Use (Link), Dako, Denmark] and EnVisionTMFLEX + (Dako, Denmark) for the visualization. The tests were carried out using Autostainer Link 48 (Dako, Denmark).

The H&E slides were scanned using UltraFast Scanner (Philips IntelliSite Solution, USA) with DigiPath™ Professional Production Software (Xerox, Norwalk, CT, USA) and representative areas of each case were selected and microtomed into 8 µm thick sections and mounted onto 1-mm-thick calcium fluoride (CaF_2_) windows (Crystran, UK).

### S-FTIR measurements and spectral analysis

The S-FTIR measurement was performed in transmission mode using a Bruker Vertex V80v FTIR spectrometer coupled with a Hyperion 2000 FTIR microscope (Bruker Optik GmbH, Ettlingen, Germany) equipped with a liquid nitrogen-cooled narrow-band mercury cadmium telluride (MCT) detector, at the Australian Synchrotron IR Microspectroscopy Beamline (Victoria, Australia). The spectral acquisition was performed using a 36× IR objective (NA = 0.50; Bruker Optik GmbH, Ettlingen, Germany) with the aperture size adjusted to 6.9 µm diameter beam size, and the spectra were acquired at a 4 µm step interval between pixels. The S-FTIR transmission maps were then acquired to cover an area of 200 μm × 200 μm on the MCL tissue. For each pixel, the S-FTIR spectrum was recorded within a spectral range of 3800–700 cm^−1^ using 4-cm^−1^ spectral resolution and 8 co-added scans. In all cases, Blackman-Harris 3-Term apodization, Power-Spectrum phase correction, and zero-filling factor of 2 were set as default acquisition parameters using OPUS 8.0.19 software suite (Bruker). Background spectra were collected from sample-free clean areas on the same CaF_2_ substrate, following the acquisition of every 50 single spectra of the tissue, using 64 co-added scans and the same default parameters.

Before spectral pre-processing atmospheric compensation function (OPUS 8.0.19 software, Bruker) was applied to remove CO_2_ and water vapour interference features. FTIR spectra embedded in acquired chemical maps were extracted and pre-processed using CytoSpec™ version 1.4.02 (Cytospec Inc., Boston, MA, USA) as follows. Prior to Hierarchical Cluster Analysis (HCA), the spectra were quality screened based upon a minimum signal-to-noise (S/N) ratio of 100 measured over the spectral ranges of 1720–1495 cm^−1^, 1440–1400 cm^−1^ and 1360–1160 cm^−1^. Next, quality-screened spectra were pre-processed using noise-reduction algorithm, followed by second derivatization using 13-point Savitzky-Golay algorithm to eliminate the broad baseline offset and curvature and to enhance the features of hidden and overlapping bands. Subsequently, spectra were vector-normalised to account for pathlength differences between samples. HCA based on five clusters was applied on the pre-processed spectra using three spectral regions of 1720–1495 cm^−1^, 1440–1400 cm^−1^ and 1360–1160 cm^−1^ to exclude the paraffin bands typically found at 2920 cm^−1^, 2850 cm^−1^, 1470 cm^−1^ and 1465 cm^−1^ [10]. As a result, obtained average absorbance and second derivative spectra were used for cluster selection for further analysis.

Principal component analysis (PCA) was performed using The Unscrambler® 10.4 software package (CAMO Software AS, Oslo, Norway). Prior to PCA selected representative second derivative spectra were corrected using Extended Multiplicative Scatter Correction (EMSC) in order to correct spectral artefacts commonly found in FTIR spectra of biological samples. The PCA approach was first applied to three individual groups: healthy control, classic MCL and aggressive MCL to eliminate outliers from samples in the same group.

After the selection of representative spectra, the EMSC-normalised second derivative spectral datasets of all groups were combined into one single set. PCA was subsequently performed on the entire combined dataset to investigate similarities and differences of biochemical makeups between healthy and malignant tissues. To exclude the bands associated with paraffin, the PCA was calculated using three spectral regions of 1720–1495 cm^−1^, 1440–1400 cm^−1^ and 1360–1160 cm^−1^.

## Supplementary information

Dataset 1

## Data Availability

For original data, please contact izazawlik@yahoo.com.
