# Publisher Correction: Classification of aggressive and classic mantle cell lymphomas using synchrotron Fourier Transform Infrared microspectroscopy

**DOI:** 10.1038/s41598-020-71092-w

**Published:** 2020-08-24

**Authors:** Magdalena Kolodziej, Dorota Jesionek-Kupnicka, Marcin Braun, Vitaliy Atamaniuk, Sylwia Sloniec, Jozef Cebulski, Marian Cholewa, Janusz Kopczynski, Philip Heraud, Mark J. Tobin, Jitraporn Vongsvivut, Izabela Zawlik

**Affiliations:** 1 Faculty of Medicine, University of Rzeszow, Rzeszow, Poland; 2 Department of Pathology, Chair of Oncology, Medical University of Lodz, Lodz, Poland; 3 Postgraduate School of Molecular Medicine, Medical University of Warsaw, Warsaw, Poland; 4 Faculty of Mathematics and Natural Sciences, University of Rzeszow, Rzeszow, Poland; 5 Department of Pathology, Holy Cross Cancer Center, Kielce, Poland; 6 Department of Microbiology and Monash Biomedical Discovery Institute, Faculty of Medicine, Nursing and Health Sciences, Monash University, Victoria, Australia; 7 Centre for Biospectroscopy, School of Chemistry, Monash University, Clayton, Australia; 8 Australian Synchrotron, ANSTO, Clayton, Victoria Australia; 9 Centre for Innovative Research in Medical and Natural Sciences, Faculty of Medicine, University of Rzeszow, Rzeszow, Poland; 10 Department of Genetics, Institution of Experimental and Clinical Medicine, University of Rzeszow, Rzeszow, Poland

Correction to: *Scientific Reports* 10.1038/s41598-019-49326-3, published online 06 September 2019

The original version of this Article contained a typographical error in the spelling of the author Vitaliy Atamaniuk, which was incorrectly given as Vitaliy Atamanyuk. This has now been corrected in the PDF and HTML versions of the Article.

