# Supplementary material for: Classification of aggressive and classic mantle cell lymphomas using synchrotron Fourier Transform Infrared microspectroscopy
Source: Sci Rep. 2019 Sep 6;9:12857. doi: 10.1038/s41598-019-49326-3 (PMC6731317; doi:10.1038/s41598-019-49326-3)
Supplement: Supplementary file 1 — Dataset 1 [file 41598_2019_49326_MOESM1_ESM.pdf]

SUPPLEMENTARY FIGURES

"Classification of aggressive and classic mantle cell lymphomas using synchrotron Fourier Transform Infrared microspectroscopy"

Authors: Magdalena Kolodziej1,+, Dorota Jesionek-Kupnicka2,+, Marcin Braun2,3,+, Vitaliy Atamaniuk4, Sylwia Sloniec4, Jozef Cebulski4, Marian Cholewa4, Janusz Kopczynski5, Philip Heraud6,7, Mark J. Tobin8, Jitraporn Vongsvivut8, and Izabela Zawlik9,10,*

[1]Faculty of Medicine, University of Rzeszow, Poland
[2]Department of Pathology, Chair of Oncology, Medical University of Lodz, Lodz, Poland
3Postgraduate School of Molecular Medicine, Medical University of Warsaw, Warsaw, Poland
4Faculty of Mathematics and Natural Sciences, University of Rzeszow, Rzeszow, Poland
5Department of Pathology, Holy Cross Cancer Center, Kielce, Poland
6Department of Microbiology & Monash Biomedical Discovery Institute, Faculty of Medicine, Nursing and Health Sciences, Monash University, Victoria, Australia
7Centre for Biospectroscopy, School of Chemistry, Monash University, Clayton, Australia
8Australian Synchrotron, ANSTO, Clayton, Victoria, Australia
9Centre for Innovative Research in Medical and Natural Sciences, Faculty of Medicine, University of Rzeszow, Rzeszow, Poland
10Department of Genetics, Institution of Experimental and Clinical Medicine, University of Rzeszow, Rzeszow, Poland
*corresponding.author: izazawlik@yahoo.com
+these authors contributed equally to this work

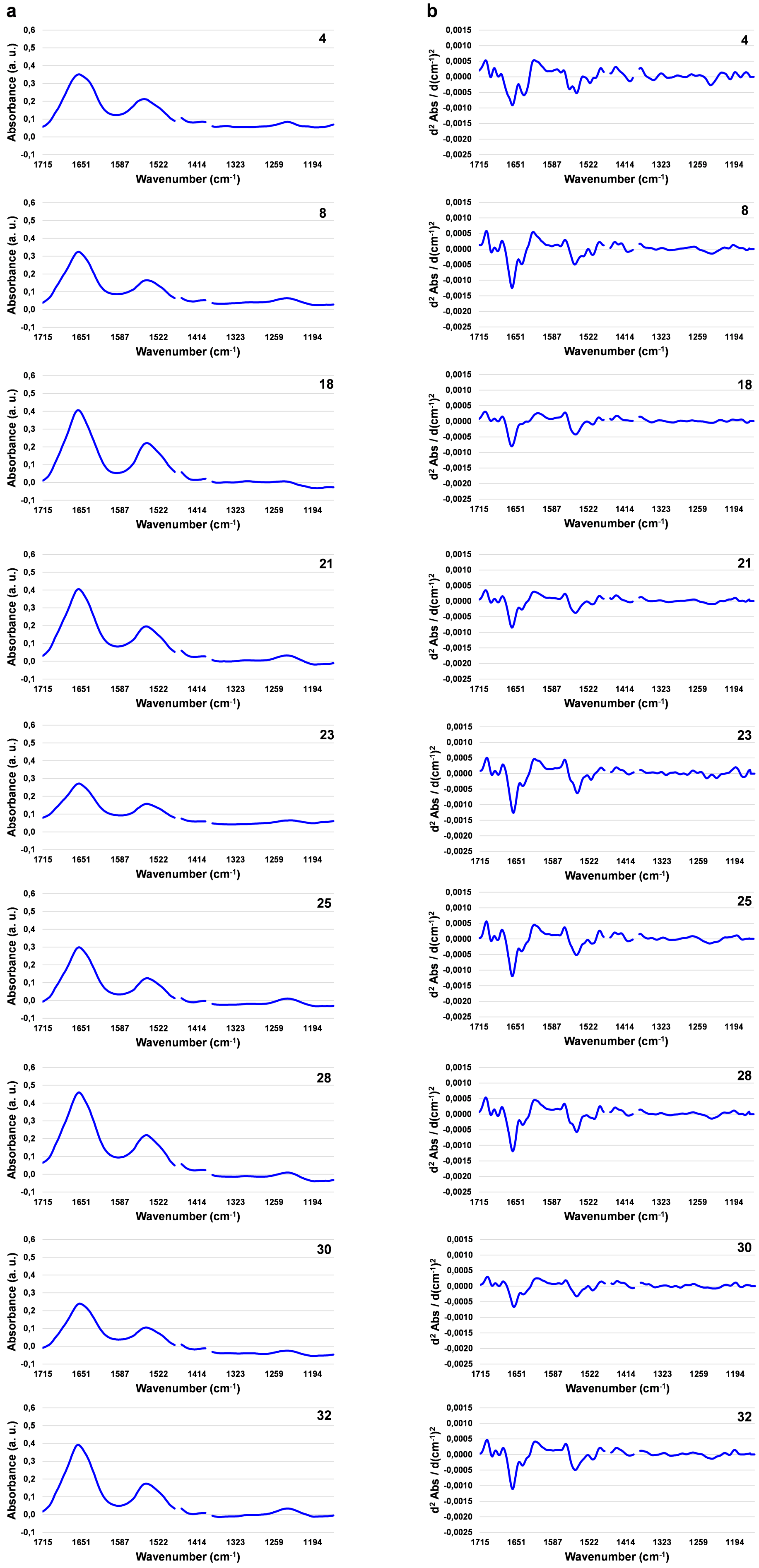

Figure S1 Average absorbance (a) and EMSC-corrected second derivative (b) spectra of the classcic mantle cell lymphoma.

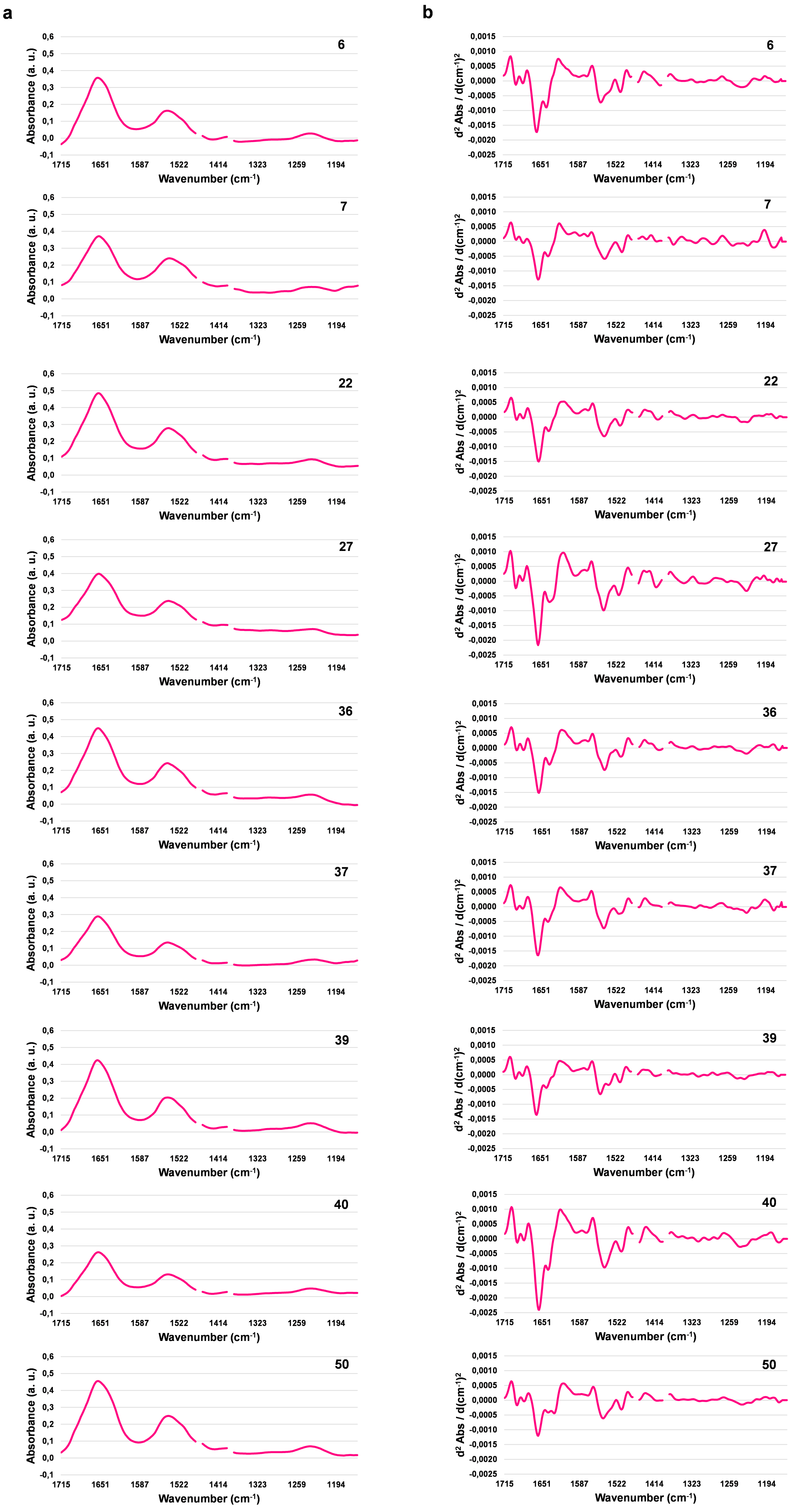

Figure S2 Average absorbance (a) and EMSC-corrected second derivative (b) spectra of the aggressive mantle cell lymphoma.

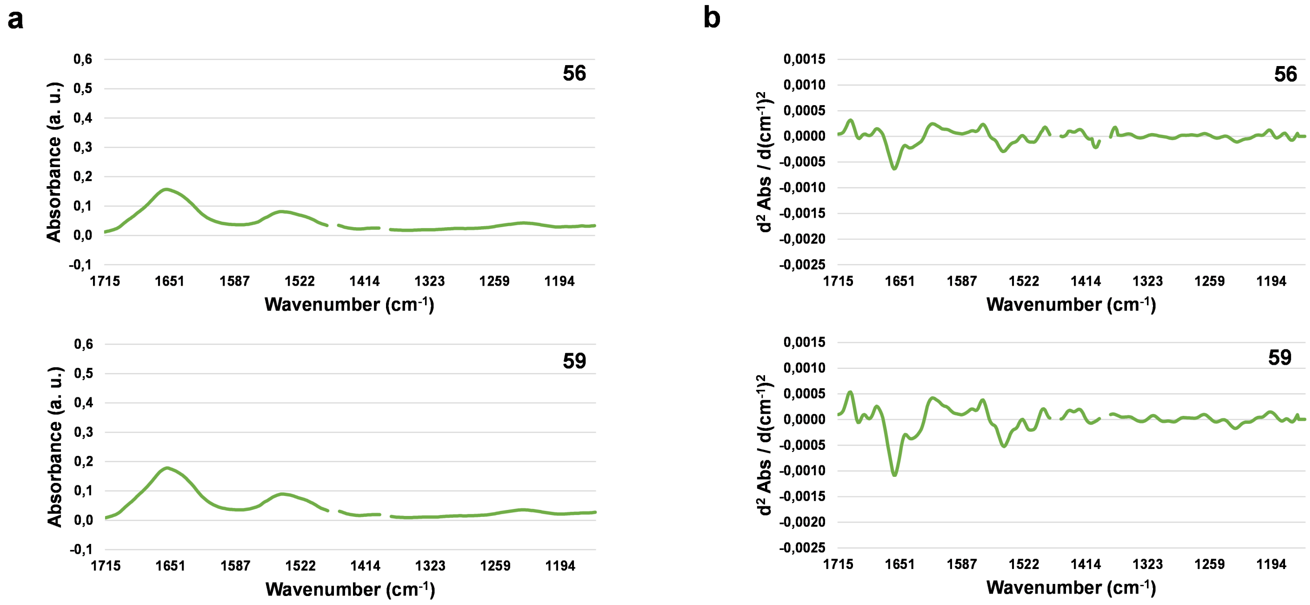

Figure S3 Average absorbance (a) and EMSC-corrected second derivative (b) spectra of the healthy control.

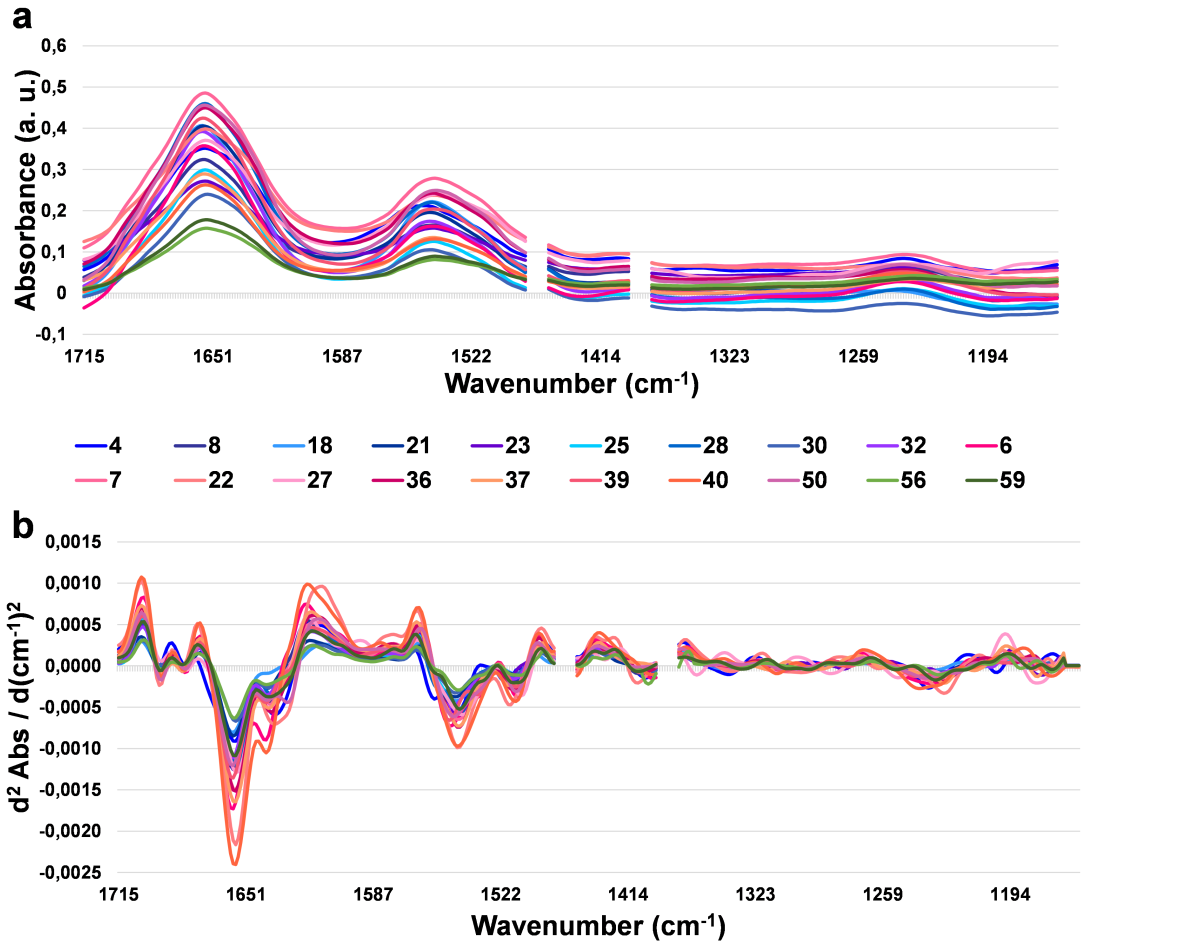

Figure S4 Average absorbance (a) and EMSC-corrected second derivative (b) spectra of healthy control and malignant tissues: classic and aggressive MCL.
